# LncRNA ZEB1-AS1 Regulates Colorectal Cancer Cells by MiR-205/YAP1 Axis

**DOI:** 10.1515/med-2020-0026

**Published:** 2020-03-08

**Authors:** Zhong Jin, Bing Chen

**Affiliations:** 1 Department of Leader/VIP Surgery, the First Affiliated Hospital of Xinjiang Medical University, No.137, South Liyushan Rd, Urumqi, 830054, Xinjiang, China; 2 Department of Gastrointestinal surgery, the First Affiliated Hospital of Xinjiang Medical University, Xinjiang, China

**Keywords:** ZEB1-AS1, miR-205, YAP1, Colorectal cancer, Proliferation, Apoptosis

## Abstract

**Background:**

Recent studies demonstrated that long non-coding RNAs (lncRNAs) were involved in many biological processes. Dysregulated lncRNAs are related to many cancers, including colorectal cancer (CRC). However, the molecular mechanism of lncRNA ZEB1-AS1 in CRC is not clear.

**Methods:**

LncRNA ZEB1-AS1, miR-205, and YAP1 expression were measured by quantitative reverse transcriptase PCR (QRT-PCR). YAP1 protein expression was measured by western blotting. Cell viability was measured by MTT assay. Cell apoptosis was detected by flow cytometry. Luciferase reporter assay was used to confirm the relationship between ZEB1-AS1, miR-205, and YAP1.

**Results:**

LncRNA ZEB1-AS1 and YAP1 was upregulated in CRC tissues. The expression of YAP1 was positively correlated with ZEB1-AS1. Knockdown of ZEB1-AS1 inhibited cell viability and induced apoptosis in CRC cell line SW480 and HCT116 which could be reversed by overexpression of YAP1. ZEB1-AS1 targeted and regulated miR-205 which could directly bind to YAP1. Meanwhile, ZEB1-AS1 regulated the expression of YAP1 via modulating miR-205.

**Conclusion:**

Long non-coding RNA ZEB1-AS1 silencing could inhibit cell proliferation and induce apoptosis of colorectal cancer via regulating miR-205 and YAP1.

## 1 Introduction

Colorectal cancer (CRC) is the development of cancer from the colon or rectum. It is the third most common cancer and the third leading cause of cancer-related death in the world [1]. In China, it was estimated that the incidence of CRC was 376,300 and the mortality was 191,000 in 2015 [2]. The initiation and progression of CRC is a complicated process involving environmental and genetic risks. Despite recent advances in understanding the underlying mechanism of CRC development, the overall survival of this malignancy remains poor because of the recurrence and metastasis [3]. Therefore, new molecular biomarkers and therapeutic targets are needed to be revealed.

Long non-coding RNAs (lncRNAs), which are mainly transcribed by RNA polymerase II, are a group of non-protein-coding RNAs. According to the size, lncRNAs are defined as transcripts which are more than 200 nucleotides in length [4]. Previous studies have demonstrated that lncRNAs are able to regulate gene expression and chromatin structure at epigenetic, transcriptional and posttranscriptional levels [4]. They are an important class of genes involved in a variety of biological processes, such as cell development [5], differentiation [6], stem cell pluripotency [7], and etc. Recently, increasing evidence indicate that many lncRNAs are aberrantly expressed in a variety of human diseases including cancers [8]. In cancers, some lncRNA can act as oncogenes or suppressors to initiate or inhibit the development of cancer, including cell proliferation, invasion, and metastasis [9]. Most important, lncRNAs can be used as tumor biomarkers and potential therapeutic targets for diagnosis and treatment of cancer [10].

Long non-coding RNA ZEB1-AS1 was firstly discovered in human hepatocellular carcinoma (HCC) in 2015 [11]. In HCC, ZEB1-AS1 was upregulated especially in metastatic tumor tissues due to the promoter hypomethylation. Patients with ZEB1-AS1 overexpression have poor recurrence-free survival. In addition, ZEB1-AS1 promoted cell proliferation and metastasis which acted as an oncogene in HCC [11]. Subsequently, ZEB1-AS1 was reported to correlate with bladder cancer [12], prostate cancer [13], and gastric cancer [14]. However, the relationship between ZEB1-AS1 and colorectal cancer is rarely reported.

In the present study, we identified that long non-coding RNA ZEB1-AS1 was upregulated in CRC tissues and cell lines SW620, SW480, HT29 and HCT116. ZEB1-AS1 silencing inhibited cell proliferation and induced apoptosis in SW480, and HCT116 cells. Thus, we hypothesized that ZEB1-AS1 plays an oncogenic role in CRC. Thus, this study was aimed to explore the molecular mechanism of ZEB1-AS1 in CRC.

## 2 Materials and Methods

### 2.1 Clinical samples

50 colorectal cancer (CRC) samples and their adjacent normal tissues were collected from the colorectal patients before undergoing chemotherapy or radiotherapy at the First Affiliated Hospital of Xinjiang Medical University. All patients read and signed the informed consent forms. All the methods in this study were in accordance with the guidelines and experimental protocols were approved by the First Affiliated Hospital of Xinjiang Medical University Research Ethics Committee.

### 2.2 Cell culture

We obtained the normal human colon mucosal epithelial cell line NCM460 and human colorectal cancer cell lines SW620, SW480, HT29, and HCT116 from American Type Culture Collection (Manassas, VA, USA). Cells were cultured in DMEM (Dulbecco’s Modified Eagle’s Medium, Gibco, USA) media with 10% fetal bovine serum (FBS, Gibco, USA), 1% penicillin and streptomycin (Sigma-Aldrich, USA) at 37°C in cell incubator with 5% CO_2_.

### 2.3 Quantitative real-time PCR (qRT-PCR)

Total RNA was extracted from CRC tissues, normal tissues, SW480, or HCT116 cells using TRIzol (ThermoFisher, USA) following the manufacturer’s instruction. QuantiTect Reverse Transcription Kit (Qiagen) was used to transcribe the cDNA for lncRNA and mRNA. MiScript II RT Kit was used to transcribe the microRNA. SYBR® Green (Promega, USA) was used to detect the expression of ZEB1-AS1, YAP1, and miR-205. U6 small nuclear RNA (snRNA) was used to normalize miR-205.18S rRNA was used to normalize ZEB1-AS1 and YAP1. QuantStudio™ 3 Real-Time PCR Systems (ThermoFisher, USA) was used to detect the fluorescence. The relative expression of all the expression were calculated by the 2^-ΔΔ^Ct method. Primer sequences: ZEB1-AS1, forward 5’- AACCTTGTTGCTAGGGACCG-3’ and reverse 5’- AGTCACTTCCCATCCCGGTT-3’; miR-205, forward 5’-CTTGTCCTTCATTCCACCGGA-3’ and reverse 5’- TGCCGCCTGAACTTCACTCC-3’; YAP1 forward 5’- TAGCCCTGCGTAGCCAGTTA-3’ and reverse 5’- TCATGCTTAGTCCACTGTCTGT-3’; 18S rRNA, forward 5’- GGCCCTGTAATTGGAATGAGTC-3’ and reverse 5’- CCAAGATCCAACTACGAGCTT-3’; U6 forward 5’-GTTGACATCCGTAAAGACC-3’ and reverse 5’-GGAGCCAGGGCAGTAA-3’.

### 2.4 Transient transfection

si-ZEB1-AS1-1, si-ZEB1-AS1-2, pcDNA-ZEB1-AS1, pcD-NA-YAP1, and miR-205 were purchased or constructed from GenePharma Co., Ltd. (Shanghai, China). All the oligos or plasmids were transfected into SW480 or HCT116 cells using Lipofectamine 3000 (Invitrogen, USA) according to the manufacturer instructions.

### 2.5 Western blot

Protein was extracted with RIPA lysis buffer (Thermo Fisher Scientific, USA) following the protocol. 20μg of each proteins sample was separated using SDS-PAGE and then transferred to polyvinylidene fluoride membranes (PVDF, Millipore, Bedford, MA, USA). Blocked with 5% dried non-fat milk in TBST (Tris-buffered saline and 0.1% Tween 20 buffe), the membranes were incubated with specific primary antibodies including anti-YAP1 (ab52771, Abcam, 1:100) and anti-GAPDH (CST#5174, 1:1000) antibody at 4°C overnight. The PVDF membrane was washed three times for 10 min each with Tris-buffered saline with 0.1% Tween-20 (TBST), following incubation with HRP-conjugated IgG secondary antibody (1:2000 dilution, Santa Cruz Biotechnology Inc.) at room temperature for 1hr. After washed with TBST three times, the protein signals were detected using Pierce^TM^ ECL western blotting substrate (ThermoFisher Scientific).

### 2.6 MTT assay

Cell viability was measured with MTT assay kit (Sigma-Aldrich, USA) according to the manufacturer’s protocol. In brief, cells (2x10^3^) were seeded into 96-well plates (Corning, USA). 100μL fresh media and 10μL MTT solution (12mM MTT solution) was added into each well and incubated for 4h at 37°C. Then discarded the medium and added 150μL dimethyl sulfoxide (DMSO, Sigma-Aldrich) into each well and incubated for 4h at 37°C. The absorbance (A) value of each well was read at OD=490nm using the Microplate Reader (MG LABTECH, Durham, NC, USA).

### 2.7 Cell apoptosis

The transfected SW480 and HCT116 were trypsinized and made into a single-cell suspension. Then washed with PBS for three times. Collected 5x10^5^ cells by centrifugation and resuspended the ells with 500ul of 1x binding buffer. Then added 5uL of annexin V-FITC and 5uL of propidium iodide (PI). Incubated the cells at room temperature for 30 min in the dark. Then observed the cells under flow cytometry.

### 2.8 Luciferase assay

SW480 or HCT116 cells were cultured in 96-well plates and co-transfected with 50 nM miR-205 (or miR-control), 50 ng of luciferase reporter vector containing WT-ZEB1-AS1, MUT-ZEB1-AS1, WT-3’UTR-YAP1, or MUT-3’UTR-YAP1 using Lipofectamine 3000. Forty-eight hours after transfection, the luciferase activities were assayed using the Dual-Luciferase Reporter Assay System (Promega, USA).

### 2.9 Statistical Analysis

The data were displayed as mean ± SD (standard deviation) from three biological replicated experiments or as indicated. The results were analyzed using GraphPad Prism 7.0 (GraphPad Software, San Diego, CA, USA). Student t-test was used to analyze the group comparison. The p values less than 0.05 was considered as statistically significant.

## 3 Results

### 3.1 ZEB1-AS1 and YAP1 was up-regulated in human colorectal cancer tissues

**Figure 1:**
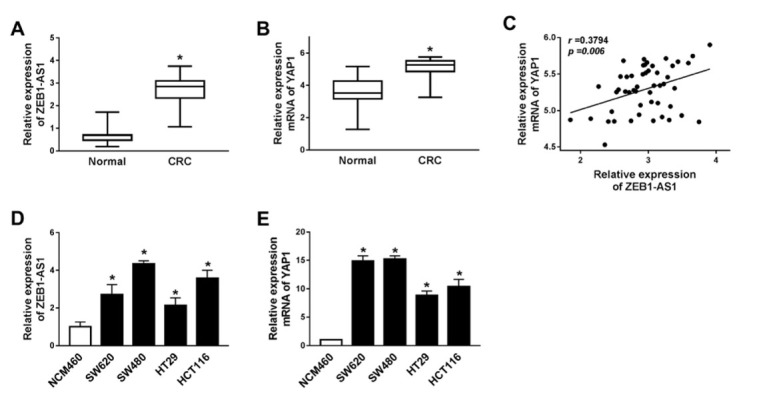
The expression level of ZEB1-AS1 and YAP1 was up-regulated in human colorectal cancer (A) ZEB1-AS1 mRNA level in CRC patients (n=50). (B) The mRNA level of YAP1 in CRC patients (n=50). (C) The correlation between ZEB1-AS1 and YAP1 in CRC patients (n=50). (D-E) ZEB1-AS1 (D) and YAP1 (E) expression in normal human colon mucosal epithelial cell line NCM460 and human colorectal cancer cell lines SW620, SW480, HT29 and HCT116. * means p < 0.05.

Long non-coding RNA ZEB1-AS1 expression was evaluated in 50 colorectal cancer samples and matched normal tissues. Quantitative reverse transcription-polymerase chain reaction (qRT-PCR) results demonstrated that ZEB1-AS1 was increased to 3 folds in CRC tumor tissues compared to normal samples (Figure 1A). Then YAP1 mRNA level was assessed and it was also upregulated in CRC tissues (Figure 1B). We also analyzed the relationship between ZEB1-AS1 and YAP1, and found that YAP1 expression was positively correlated with ZEB1-AS1 in CRC tissues (Figure 1C, r=0.3794, p=0.006). Next, we measured the expression of ZEB1-AS1 in human colon mucosal epithelial cell line NCM460 and human colorectal cancer cell lines SW620, SW480, HT29, and HCT116 and found that ZEB1-AS1 was significantly upregulated in CRC cell lines (Figure 1D). Consistently, the expression of YAP1 was extremely higher in CRC cell lines (SW620, SW480, HT29, HCT116), especially in SW480 and HCT116 cells, than that of human colon mucosal epithelial cell line NCM460 (Figure 1E). Thus, we chose SW480 and HCT116 for the next experiments.

### 3.2 Knockdown of ZEB1-AS1 inhibits proliferation and induced apoptosis of CRC cells

**Figure 2:**
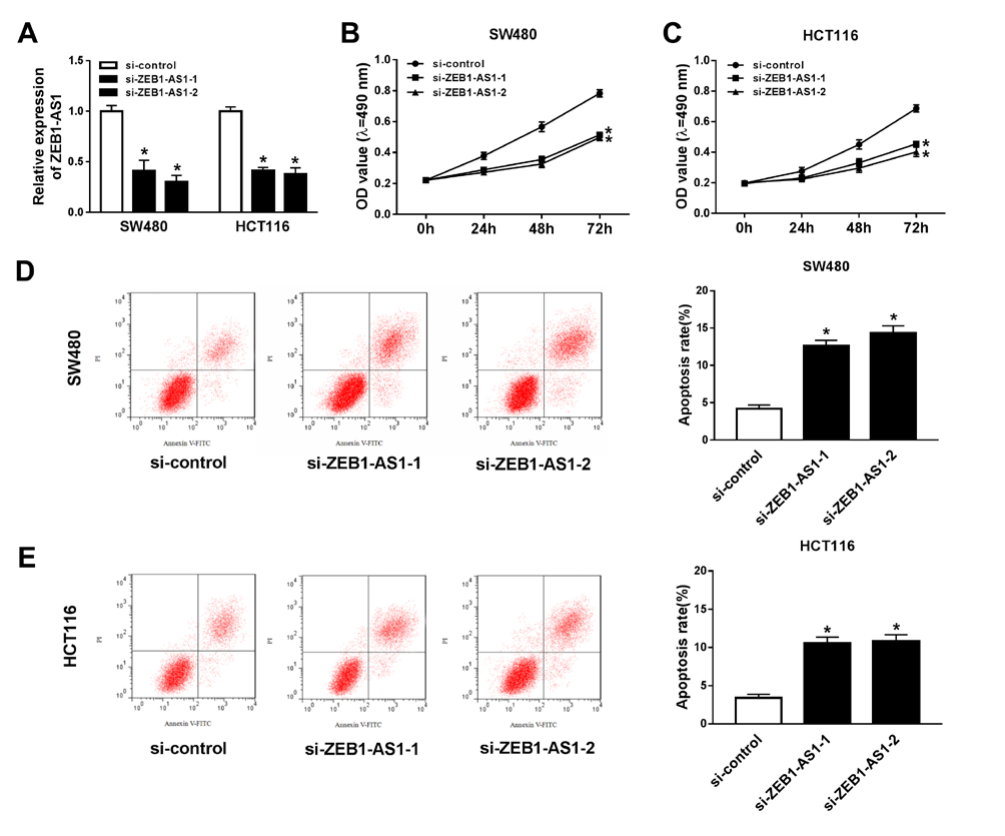
Knockdown of ZEB1-AS1 inhibits proliferation and apoptosis of CRC cells (A) ZEB1-AS1 siRNA knockdown efficiency in SW480 and HCT116 cells. (B) The proliferation curve of SW480 after ZEB1-AS1 knockdown. (C) The proliferation of HCT116 after ZEB1-AS1 knockdown. (D) Apoptotic rate of SW480 after ZEB1-AS1 knockdown. (E) Apoptotic rate of HCT116 after ZEB1-AS1 knockdown. Annexin V positive cells were considered as apoptotic cells.* means *p* < 0.05.

To better understand the role of ZEB1-AS1 in CRC, we knocked down the expression of ZEB1-AS1 using small RNA interfere in SW480 and HCT116 CRC cells. After screening, we chose 2 siRNAs, si-ZEB1-AS1-1, and si-ZEB1-AS1-2. Both of the si-ZEB1-AS1-1 and si-ZEB1-AS1-2 induced striking reduction of ZEB1-AS1 expression in SW480 and HCT116 cells (Figure 2A). Next, cell proliferation and apoptosis effected by ZEB1-AS1 knockdown was examined in transfected SW480 and HCT116 cells. MTT assay suggested that cell proliferation was decreased significantly after ZEB1-AS1 knockdown in SW480 (Figure 2B) and HCT116 cells (Figure 2C). Then SW480 cells were costained with Annexin V and propidium iodide (PI) after ZEB1-AS1 knockdown. The apoptotic cells which were Annexin V positive increased from 4.21±0.50% to 12.64±0.72% or 14.32±0.98% (Figure 2D). Also, in HCT116 cells, the apoptotic rate increased from 3.41±0.46% to 10.57±0.78% or 10.82±0.86% (Figure 2E). The results indicated that knockdown of ZEB1-AS1 inhibited cell proliferation and induced cell apoptosis in SW480 and HCT116 cells.

### 3.3 YAP1 overexpression reversed effects of ZEB1-AS1 knockdown in proliferation and apoptosis

**Figure 3:**
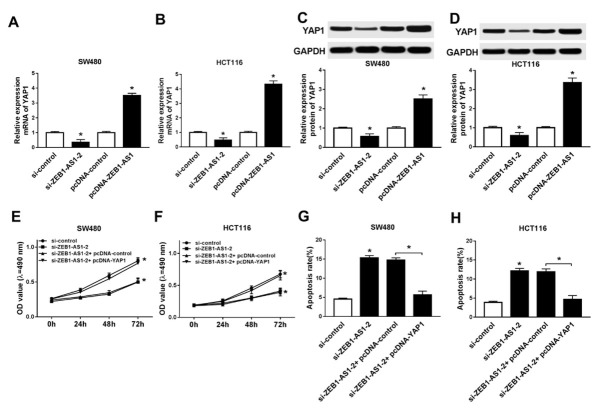
YAP1 overexpression reversed effects of ZEB1-AS1 knockdown on proliferation and apoptosis of CRC cells (A) The mRNA expression of YAP1 in SW480 cell after ZEB1-AS1 knockdown and overexpression. (B) The mRNA expression of YAP1 in HCT116 cell after ZEB1-AS1 knockdown and overexpression. (C) The protein level of YAP1 in SW480 cell after ZEB1-AS1 knockdown and overexpression. (D) The protein level of YAP1 in HCT116 cell after ZEB1-AS1 knockdown and overexpression. (E) Cell proliferation of SW480 after ZEB1-AS1 knockdown and YAP1 overexpression. (F) The proliferation of HCT116 after ZEB1-AS1 knockdown and YAP1 overexpression. (G) Cell apoptosis of SW480 after ZEB1-AS1 knockdown and YAP1 overexpression. (H) Cell apoptosis of HCT116 after ZEB1-AS1 knockdown and YAP1 overexpression.* means *p* < 0.05.

To further explore the relationship between ZEB1-AS1 and YAP1, we knocked down and overexpressed ZEB1-AS1 in SW480 and HCT116 cells. YAP1 mRNA levels were significantly decreased in SW480 and HCT116 cells after ZEB1-AS1 knockdown, while YAP1 expression was upregulated after ZEB1-AS1 overexpression (Figure 3A and 3B). Consistently, YAP1 protein was downregulated after ZEB1-AS1 knockdown and upregulated when overexpressed ZEB1-AS1 in SW480 and HCT116 cells (Figure 3C and 3D). Next, we knocked down ZEB1-AS1 and overexpressed YAP1 in SW480 and HCT116 cells simultaneously. The cell proliferation was inhibited by knockdown of ZEB1-AS1, whereas ectopic expression of YAP1 abolished these effects in SW480 (Figure 3E) and HCT116 cells (Figure 3F). Moreover, YAP1 overexpression reversed promotion of ZEB1-AS1 knockdown in apoptosis in SW480 (Figure 3G) and HCT116 cells (Figure 3H). The results demonstrated that YAP1 was regulated by ZEB1-AS1 and overexpression of YAP1 reversed the effect of ZEB1-AS1 knockdown in cell proliferation and apoptosis.

### 3.4 ZEB1-AS1 directly interacted with miR-205

**Figure 4:**
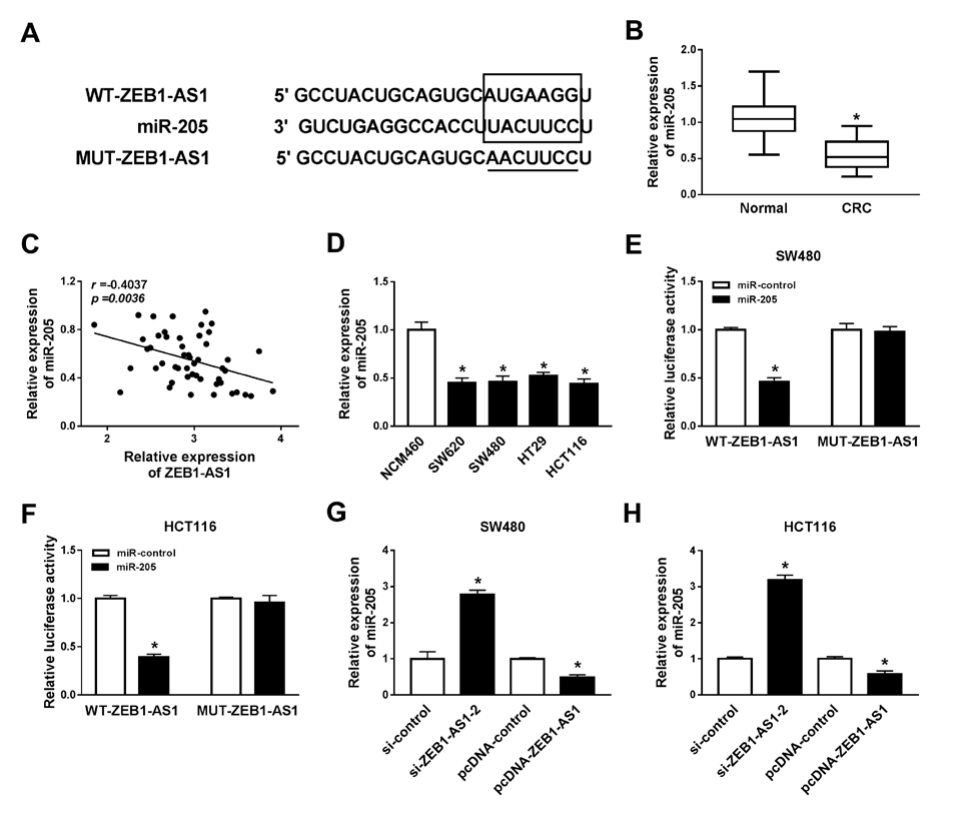
ZEB1-AS1 directly interacts with miR-205 (A) The relationship between ZEB1-AS1 and miR-205 predicted by MiRcode Tools. (B) The level of miR-205 in CRC patients (n=50). (C) The correlation between ZEB1-AS1 and miR-205 in CRC patients (n=50). (D) The expression of miR-205 in normal human colon mucosal epithelial cell line NCM460 and human colorectal cancer cell lines SW620, SW480, HT29 and HCT116. (E) Luciferase assay of WT-ZEB1-AS1/MUT-ZEB1-AS1 and miR-205 in SW480 cells. (F) Luciferase assay of WT-ZEB1-AS1/MUT-ZEB1-AS1 and miR-205 in HCT116 cells. (G) MiR-205 expression in SW480 cells after silencing or overexpression of ZEB1-AS1. (H) MiR-205 expression in HCT116 cells after silencing or overexpression of ZEB1-AS1. * means *p* < 0.05.

Recent studies proposed that lncRNAs can act as miRNA sponges to exert their function [15]. Thus, MiRcode Tools was used to predict the potential target miRNA. As shown in Figure 4A, miR-205 had complementary sequences with ZEB1-AS1. To explore the role of miR-205 in CRC cell growth, the expression of miR-205 was detected by QRT-PCR. As illustrated in Figure 4B, the level of miR-205 was down-regulated in CRC tumor tissues compared with normal tissues. Similarly, miR-205 expression was relatively lower in CRC cell lines than that of NCM460 cells (Figure 4D). Person’s correlation coefficient analysis revealed that ZEB1-AS1 was correlated with miR-205 inversely (r=-0.4037, p=0.0036) (Figure 4C). To validate the interaction of ZEB1-AS1 and miR-205, we constructed a luciferase reporter vector containing wild-type ZEB1-AS1 (WT-ZEB1-AS1) or mutant ZEB1-AS1 (MUT-ZEB1-AS1). We cotransfected the luciferase reporter vector and miR-205 into SW480 and HCT116 cells. The luciferase activity was decreased when cotransfected with WT-ZEB1-AS1 and miR-205 in SW480 and HCT116 cells, but no changes with MUT-ZEB1-AS1 (Figure 4E and 4F). Next, we measured the miR-205 expression when knocking down or overexpressing ZEB1-AS1. Obviously, miR-205 was upregulated while ZEB1-AS1 was knocked down in SW480 and HC116 cells and downregulated while ZEB1-AS1 was overexpressed (Figure 4G and 4H). These results indicated that ZEB1-AS1 could target miR-205 and regulate it.

### 3.5 YAP1 was a mRNA target of miR-205

**Figure 5:**
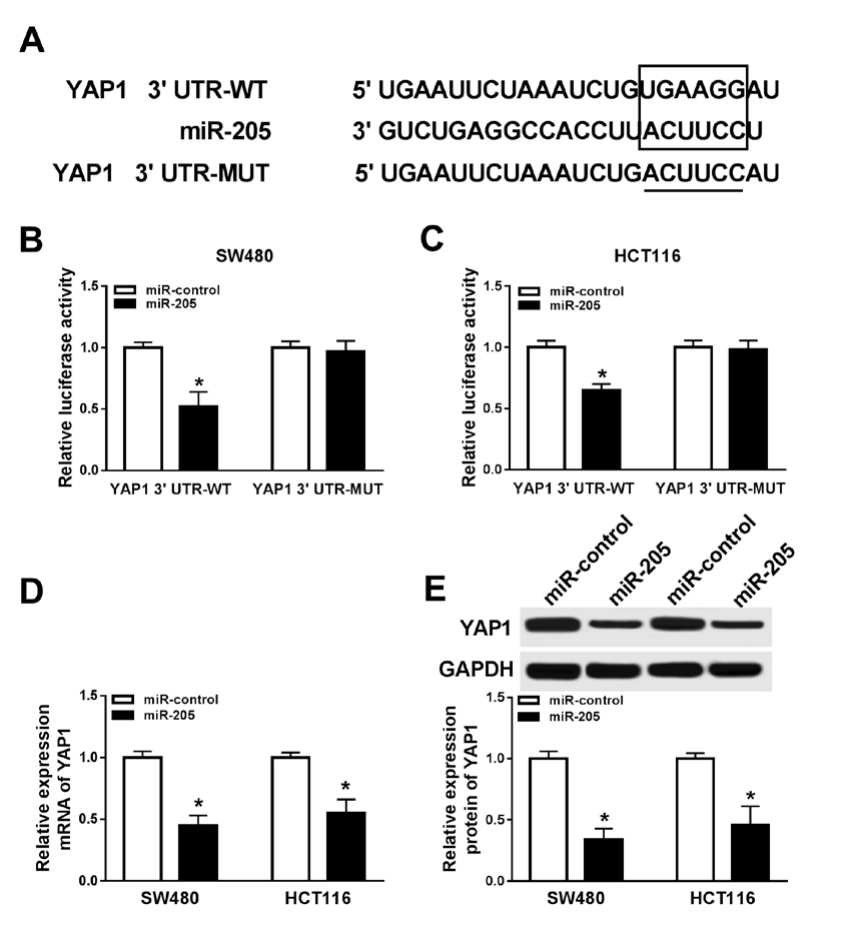
miR-205 directly interacts with YAP1 (A) The relationship between miR-205 and YAP1 predicted by TargetScan Tools. (B) Luciferase assay of WT-YAP1/MUT-YAP1 and miR-205 in SW480 cells. (C) Luciferase assay of WT-YAP1/MUT-YAP1 and miR-205 in HCT116 cells. (D) YAP1 mRNA expression in SW480 and HCT116 cells after overexpression of miR-205. (E) YAP1 protein expression in SW480 and HCT116 cells after overexpression of miR-205. * means *p* < 0.05.

Next, Targetscan Tools predicated that YAP1 was a target of miR-205. So we used luciferase assay to confirm the relationship. Similarity, we constructed a luciferase reporter vector containing wild-type YAP1 (YAP1 3’UTR-WT) or mutant YAP1 (YAP1 3’UTR-MUT) (Figure 5A). The luciferase activity was decreased when cotransfected with YAP1 3’UTR-WT and miR-205 in SW480 and HCT116 cells, but no changes with YAP1 3’UTR-MUT (Figure 5B and 5C). In addition, YAP1 mRNA levels were decreased when miR-205 was overexpressed in SW480 and HCT116 cells (Figure 5D). The protein level of YAP1 was also downregulated after miR-205 overexpression in SW480 and HCT116 cells (Figure 5E). These results suggested that miR-205 targeted and regulated YAP1.

### 3.6 ZEB1-AS1 targeted miR-205 to regulate YAP1 expression

**Figure 6:**
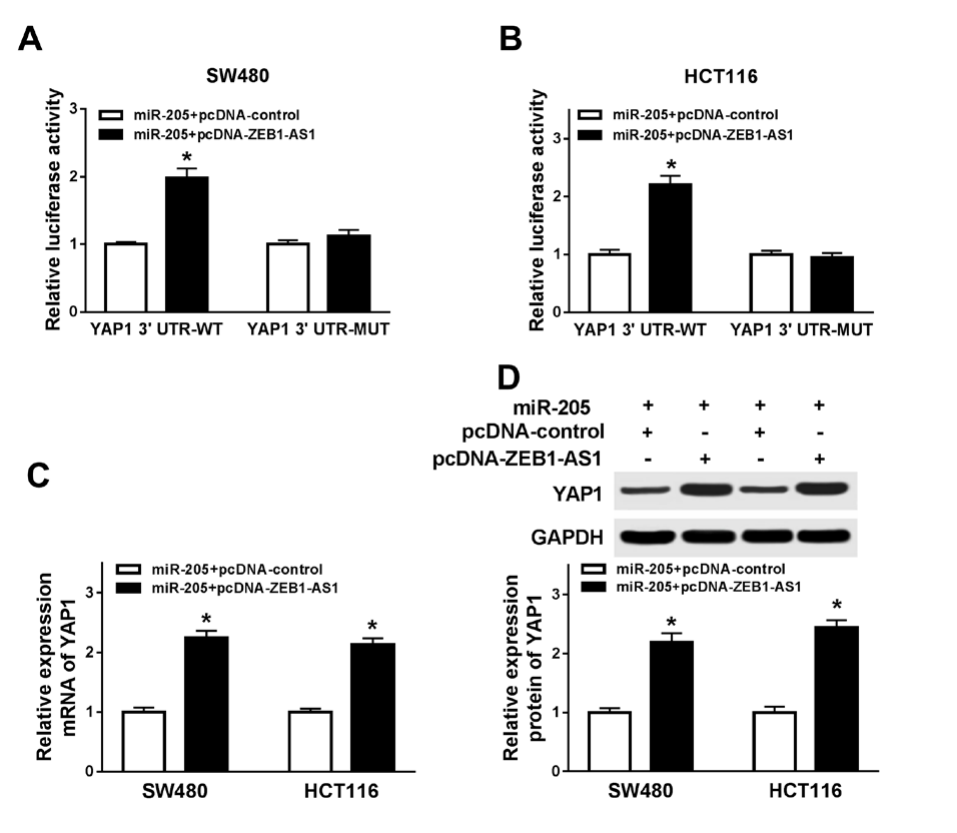
ZEB1-AS1 targeted miR-205 to regulate YAP1 expression (A) Luciferase assay of 3’UTR of YAP1, ZEB1-AS1 and miR-205 in SW480 cells. (B) Luciferase assay of 3’UTR of YAP1, ZEB1-AS1 and miR-205 in HCT116 cells. (C) mRNA level of YAP1 in SW480 and HCT116 cells after overexpression of both miR-205 and ZEB1-AS1. (D) YAP1 protein level in SW480 and HCT116 cells after overexpression of both miR-205 and ZEB1-AS1. * means p < 0.05.

To further understand the relationship between ZEB1-AS1, miR-205, and YAP1, we cotransfected luciferase reporter vector containing YAP1 3’ UTR-WT or YAP13’UTR-MUT, miR-205, pcDNA-control or pcDNA-ZEB1-AS1 into SW480 and HCT116 cells. ZEB1-AS1 overexpression reversed the inhibitory effect of miR-205 on luciferase activity with YAP1 3’UTR-WT in SW480 (Figure 6A) and HCT116 (Figure 6B) cells. Meanwhile, ZEB1-AS1 overexpression reversed the inhibitory effect of miR-205 on YAP1 mRNA level and protein level in SW480 and HCT116 cells (Figure 6C and 6D). These results indicated that ZEB1-AS1 regulated YAP1 expression via targeting miR-205.

## 4 Discussion

Emerging pieces of evidences have suggested that long non-coding RNA (lncRNAs) are related with cancers [9]. LncRNA expression is frequently increased or decreased in various cancers which are related to the development, metastasis, recurrence and poor prognosis [16]. Zinc finger E-box binding homeobox 1 antisense 1 (ZEB1-AS1) is a long coding RNA which is an antisense transcript generated from ZEB1 promoters. Meanwhile, ZEB1-AS1 is overexpressed in many cancers such as bladder cancer [12], prostate cancer [13], gastric cancer [14], esophageal cancer [17], pancreatic cancer [18], osteosarcoma [19], and breast cancer [20]. In colorectal cancer, ZEB1-AS1 also plays an important role in the development and progression. *Gong* *et al*. suggested that ZEB1-AS1 overexpression promoted cell proliferation by regulating p15 expression in CRC [21]. *Xiong et al*. proved that ZEB1-AS1 regulated the proliferation and migration of CRC cells via the miR-101/ZEB1 axis [22]. *Lv et al*. revealed that ZEB1-AS1 sponges miR-181a-5p to promote CRC proliferation by regulating Wnt/β-catenin signaling [23]. However, it is still to explore the detailed oncogenic function in CRC. In our study, we found that ZEB1-AS1 was upregulated in CRC tissues and cell lines. Knockdown of ZEB1-AS1 inhibited cell proliferation and induced apoptosis in SW480 and HCT116 cells. Through bioinformatic analysis and luciferase assay, we demonstrated that ZEB1-AS1 can target and regulate the expression of miR-205.

MicroRNAs (miRNAs) may function to regulate the expression levels of other genes mainly by inhibiting the transcription or mediating the degradation. It has been reported that miRNAs are involved in many cellular processes including development, proliferation, and apoptosis [24]. Altered expression of miRNAs are related with the initiation and progression of cancers. MiR-205 is a highly conserved microRNA among different species which locates in the second intron of LOC642587 locus on chromosome 1 in human [25]. The essential roles of miR-205 are to orchestrate the biological processes in the morphogenesis of epithelium during embryogenesis [26]. Recently, accumulating studies have indicated that miR-205 has a dual function which may act as a tumor suppressor or oncogene by targeting corresponding oncogenes in various cancer [27]. For example, in breast cancer, miR-205 was shown to suppress cell growth via directly binding to ErbB3 and VEGF-A [28]. However, miR-205 overexpression promoted cell proliferation and blood vessel formation in non-small cell lung cancer via the AKT pathway [29]. In our study, we found that miR-205 was a target of long non-coding RNA ZEB1-AS1 via bioinformatics analysis and luciferase assay. Meanwhile, miR-205 was negatively regulated by ZEB1-AS1 in CRC cells.

In the present study, we firstly certified that Yes Associated Protein 1 (YAP1) has potential binding sites with miR-205 which was confirmed by luciferase assay in CRC cells. YAP1 is a downstream nuclear effector of the Hippo signaling pathway which is involved in development, growth, repair, and homeostasis at the baseline [30]. In many cancers, overexpression of YAP1 is considered as a poor prognostic marker, such as gastric cancer [31], ovarian cancer [32]. In CRC, YAP1 also acts as an oncogene. *Liu et al*. revealed that activated YAP1 promoted colorectal cancer growth and metastasis [33]. In our study, we found that YAP1 was upregulated in CRC tissues which was positively correlated with ZEB1-AS1. In addition, overexpression of YAP1 reversed the inhibitory effects of ZEB1-AS1 silencing in cell proliferation and decreased cell apoptosis in CRC cells. However, the details of the YAP1 function in tumor cells is needed to be explored in future studies.

## 5 Conclusion

We have identified that long non-coding RNA ZEB1-AS1 was upregulated in CRC tissues as well as in CRC cell line SW620, SW480, HT29, and HCT116. Meanwhile, protein-coding RNA YAP1 was also upregulated in CRC tissues and cells which was positively correlated with ZEB1-AS1. Then we used RNA interfere to knock down the expression of ZEB1-AS1 in CRC cells and found that ZEB1-AS1 silencing inhibited cell proliferation and induced apoptosis which could be reversed by YAP1 overexpression. Next, we found ZEB1-AS1 could target miR-205 and regulate its expression while miR-205 targeted and regulated YAP1 expression in CRC cells. Meanwhile, ZEB1-AS1 could competitively bind to miR-205 and regulate YAP1 expression via miR-205. In summary, our findings revealed that the ZEB1-AS1/miR-205/YAP1 axis plays an important role in colorectal cancer which provides potential molecular biomarkers and therapeutic targets in CRC.
